# Impact of cholesterol homeostasis within cochlear cells on auditory development and hearing loss

**DOI:** 10.3389/fncel.2023.1308028

**Published:** 2024-01-04

**Authors:** Jichang Wu, Peilin Ji, Andi Zhang, Haixia Hu, Yilin Shen, Quan Wang, Cui Fan, Kaili Chen, Rui Ding, Weiyi Huang, Mingliang Xiang, Bin Ye

**Affiliations:** ^1^Department of Otolaryngology and Head and Neck Surgery, Ruijin Hospital, Shanghai Jiao Tong University School of Medicine, Shanghai, China; ^2^Department of Audiology and Speech-Language Pathology, College of Health Science and Technology, Shanghai Jiao Tong University School of Medicine, Shanghai, China

**Keywords:** hypercholesterolemia, cholesterol homeostasis, hearing loss, therapeutic targets, cochlea

## Abstract

Cholesterol is the most abundant sterol molecule in mammalian cells, which not only constitutes the cell membrane but also plays essential roles in the synthesis of important hormones, synapse formation, and cell signal transduction. The effect of hypercholesterolemia on hearing has been studied extensively, and multiple studies have demonstrated that hypercholesterolemia is a risk factor for hearing loss. However, the impact of cholesterol homeostasis within auditory cells on peripheral auditory development and maintenance has not been evaluated in detail. Mutations in certain cholesterol metabolism-related genes, such as *NPC1*, *SERAC1*, *DHCR7*, and *OSBPL2*, as well as derivatives of cholesterol metabolism-related ototoxic drugs, such as β-cyclodextrin, can lead to disruptions of cholesterol homeostasis within auditory cells, resulting in hearing loss. This article aims to review the impact of cholesterol homeostasis within auditory cells on the peripheral auditory function from the following two perspectives: (1) changes in cholesterol homeostasis regulatory genes in various hearing loss models; (2) mechanisms underlying the effects of some drugs that have a therapeutic effect on hearing loss via regulating cholesterol homeostasis. This article aims to summarize and analyze the impact of disruption of cellular cholesterol homeostasis within auditory cells on hearing, in order to provide evidence regarding the underlying mechanisms.

## 1 Introduction

### 1.1 Basic concepts of cholesterol homeostasis

Cholesterol is the sterol molecule with the highest content in mammalian cells and is distributed mainly on the plasma membrane. It is abundant in the nervous system, such as the central nervous system, which accounts for only 2% of the whole body mass but contains almost a quarter of the unesterified cholesterol in the body (Dietschy and Turley, 2001). In addition to regulating membrane fluidity, cholesterol is used to synthesize steroid hormones, such as aldosterone, cortisol, sex hormones, and vitamin D. It is also involved in bile acid metabolism, which in turn affects food absorption and utilization and participates in the covalent modification of intracellular signaling factors, such as hedgehog and smoothened homolog protein.

Cholesterol homeostasis refers to the dynamic equilibrium of cholesterol absorption, synthesis, transport, and excretion, which ensures that the functions of cells, tissues, and organs are within the normal physiological range. Cholesterol homeostasis is typically divided into extracellular (intercellular transport) and intracellular (cholesterol utilization, transport, and excretion) cholesterol homeostasis. Many studies have shown that intracellular cholesterol homeostasis is essential for the normal structure and physiological function of neurons and its imbalance is related to several diseases in central nervous system (Yoon et al., 2022). In the brain, cholesterol is primarily derived from surrounding glial cells, with some being synthesized internally. Maintaining intracellular cholesterol homeostasis in order is crucial for the normal function of neurons (Pfrieger and Ungerer, 2011).

### 1.2 Regulation of intracellular cholesterol

Low-density lipoprotein (LDL) cholesterol is the primary source of cholesterol provision for neural tissues outside the brain. LDL is the main carrier for transporting endogenous cholesterol. After being recognized and bound by LDL receptors (LDLRs) on the cell membrane, it is internalized by the cell and sorted into lysosomes, where it is hydrolyzed and releases free cholesterol. The free cholesterol then binds with Niemann-Pick disease type C1 (NPC1) and Niemann-Pick disease type C2 (NPC2) proteins in the lysosomes and is transported to other organelles, such as the endoplasmic reticulum and peroxisomes, for further processing and utilization, including the synthesis of steroid hormones and bile acids (Luo et al., 2020). NPC1 is the pathogenic gene for Niemann-Pick disease, and patients with this disease often have concomitant sensorineural hearing loss (King et al., 2014b).

The genes responsible for cholesterol synthesis in cells are primarily regulated by the sterol regulatory element-binding proteins (Horton et al., 2002), which enter the nucleus of cells to activate the expression of genes related to cholesterol synthesis and LDLR, thus restoring the balance of intracellular cholesterol concentration. The main genes that regulate cholesterol efflux in cells are transcription factors called liver X receptors (LXRs) (Zhao and Dahlman-Wright, 2010).

Liver X receptor (LXR) is a cholesterol sensor within cells that regulates the cholesterol homeostasis by controlling the efflux of cholesterol, synthesis of bile acids and fatty acids, and levels of lipid transport genes. LXR has two subtypes, LXRα and LXRβ. LXRα is mainly expressed in the liver, adipose tissue, and other tissues, while LXRβ is highly expressed in the nervous system.

## 2 Role of intracellular cholesterol homeostasis in auditory function

### 2.1 Impact of dysregulated intracellular cholesterol homeostasis on auditory development

Lipid rafts are microdomains located on the cell membrane that are rich in cholesterol and sphingolipids, which are important functional units of the cell membrane. Cholesterol is an important component of lipid rafts, which stabilizes the membrane domain by interacting with other lipids and specific types of raft proteins, thereby regulating the fluidity of the cell membrane (Crane and Tamm, 2004). β-cyclodextrin is a cyclic oligosaccharide produced by amylase hydrolysis that acts as a molecular capsule. Derivatives of β-cyclodextrin, such as methyl-β-cyclodextrin and hydroxypropyl-β-cyclodextrin (HPβCD), can be used to transport drugs and modify the solubility of drugs, thereby increasing the drug release rates. β-cyclodextrin can also deplete cholesterol on the cytoplasmic lipid raft, leading to the disruption of cell membrane fluidity and integrity. Recently, Levic and Yamoah (2011) reported that adding methyl-β-cyclodextrin to the hair cells of the developing (E12) chicken basilar papilla can deplete cholesterol on their cell membranes and enhance the voltage-gated K+ ion channels of the hair cells (i.e., increases IKv). The extent of enhancement is proportional to the decrease in the cholesterol level in mature hair cell membranes. The voltage-gated K+ ion channel in developing hair cells produces the spontaneous electrical activity and is a marker for cell development and regeneration. Therefore, the cholesterol content of the membrane of the developing chicken hair cells is related to the degree of spontaneous activity and is a marker of the formation of the fine loop of mature hair cells and the auditory system.

### 2.2 Effect of intracellular cholesterol homeostasis disorder on hereditary hearing loss

Some studies have shown that disorders of cochlear cholesterol homeostasis play an important role in hereditary hearing loss. NPC1 and NPC2 are the main proteins of intracellular cholesterol transport, which can bind to cholesterol and participate in its intracellular transport. Niemann-Pick disease is an inherited neurodegenerative disorder, in which the NPC1 gene mutations result in abnormal cholesterol accumulation in lysosomes, associated with sensorineural hearing loss in some patients (King et al., 2014b). Using toluidine blue staining, King et al. (2014a) found that P20 mice with NPC1 gene homozygous knockout exhibited significant cholesterol accumulation in cochlear hair cells. Moreover, with increasing mice age, the amount of cholesterol stored in hair cells increased, resulting in worsening hearing capacity (King et al., 2014a). SERAC1 gene encodes a phospholipid glycerol remodeling protein, and its mutation leads to MEGDEL syndrome, which is characterized by 3-methylglutaconic aciduria, hearing loss, encephalopathy, and Leigh-like symptoms. Approximately 79% of patients with MEGDEL syndrome have hearing loss, and 58% never learn to speak (Maas et al., 2017). SERAC1 protein plays a major role in intracellular cholesterol transport and mitochondrial function regulation, whereas its mutation leads to intracellular cholesterol accumulation (Wortmann et al., 2012). Smith-Lemli-Opitz syndrome is an autosomal recessive disease characterized by intellectual disability and microcephaly; its pathogenic mechanism involves a mutation in the cholesterol synthase DHCR7 of neurons, resulting in abnormal accumulation of cholesterol precursor 7-dehydrocholesterol. Patients with this disease may be relieved by dietary cholesterol supplementation or statins. Approximately 65.6% of patients with this disorder have various types of hearing loss, and the incidence of sensorineural hearing loss is 21.9% (Zalewski et al., 2021), which may be related to myelin sheath developmental disorders of the auditory nerve. Oxysterol binding protein-like 2 (OSBPL2) is a newly identified autosomal dominant sensorineural hearing loss gene (Xing et al., 2015), which is mainly involved in maintaining cholesterol homeostasis by participating in cholesterol transport. In the pig model with an OSBPL2 gene mutation, the hearing threshold of 2–16 kHz was significantly increased at 2 months of age (Yao et al., 2019). OSBPL2 knockout in the auditory cell line OC1 leads to increased intracellular cholesterol synthesis and enhanced oxidative stress response, thereby promoting cell death (Wang et al., 2019). These results suggest that dysregulation of cochlear cholesterol homeostasis is an important factor involved in the development of hereditary hearing loss (Table 1).

**TABLE 1:** Disturbed cholesterol homeostasis caused by genes and drugs in hearing loss.

Gene/drug gene	Disease	Hearing loss type in human	Hearing loss in phenotype and pathology	Underlying mechanism	References
NPC1	Niemann-Pick disease, type C1	Syndromic hereditary hearing loss	A great quantity of cholesterol accumulates in cochlear hair cells of mice	Defective intracellular processing and transport of cholesterol, causes accumulation of cholesterol in lysosomes	King et al., 2014a
DHCR7	Smith-Lemli-Opitz syndrome	Syndromic hereditary hearing loss	Audiometric phenotype is heterogeneous including conductive, mixed, and sensorineural	Error of cholesterol synthesis caused by deficiency of 7-dehydrocholesterol reductase	Zalewski et al., 2021
OSBPL2	Deafness, autosomal dominant 67	non-syndromic hereditary hearing loss	Hearing loss initially affects high frequencies, with variable progression	Mediates cholesterol transport	Xing et al., 2015; Yao et al., 2019
SERAC1	MEGDEL syndrome	Syndromic hereditary hearing loss	Hearing problems gradually worsen over time	Intracellular cholesterol trafficking problem, abnormal accumulation of unesterified cholesterol within cells	Maas et al., 2017
Derivatives of β- cyclodextrin	Ototoxic drug	Sensorineural hearing loss	Priority damage to outer hair cells, possibly related to prestin	Consume the cholesterol of lipid raft, leading to the destruction of the fluidity and integrity of cell membrane	Crumling et al., 2017; Ding et al., 2021

NPC1, Niemann-Pick type C1; DHCR7, 7-dehydrocholesterol reductase; OSBPL2, oxysterol binding protein-like 2; SERAC1, serine active site-containing protein 1.

### 2.3 Role of intracellular cholesterol homeostasis disorder in noise-induced hearing loss

Previous studies on noise-induced hearing loss mainly focused on the effects of hyperlipidemia on noise susceptibility and peripheral auditory organs, with little attention paid to changes in cholesterol metabolism within the cochlea under noise exposure. Furthermore, the studies did not evaluate whether hearing-protective drugs exert their effects on noise-induced hearing loss by affecting cholesterol metabolism pathways. In Milon et al. (2021) published the results of single-cell sequencing of the inner ear of CBA/CaJ mice under noise stimulation. After exposure of adult CBA/CaJ mice (aged 2–4 months) to noise (105–110 dB, 2 h) under normal feeding conditions, single-cell sequencing of the cochlear spiral ganglions performed 24 h later revealed that the main transcriptional regulatory factors upstream of differentially expressed genes in type Ia spiral ganglions before and after noise exposure were ATF3/4 (upregulated) and SREBF1 (downregulated). Furthermore, using the results of whole cochlear single-cell sequencing through DrugCentral drug screening, statins were found to be the top-ranked drug for the treatment of noise-induced hearing loss (Milon et al., 2021). In Sai et al. (2020) first reported the model of hearing loss in miniature pigs under acute noise exposure (120 dB, white noise). They performed cochlear proteomic analysis and found that, during the recovery stage of acute hearing loss, most differentially expressed proteins were related to cholesterol metabolism, among which the lipoproteins ApoAI and ApoE were significantly upregulated (Sai et al., 2020). These studies confirmed that the cholesterol metabolism pathways are involved in the development of noise-induced hearing loss at the genetic and protein levels. ChIP analysis of the cochlea of mice with noise-induced hearing loss showed that LDLR was a hub gene for the occurrence of noise-induced hearing loss (Wang et al., 2020).

Metformin is the first-line drug for the treatment of type 2 diabetes, and it plays an important role in neuroprotection. Few studies have reported that metformin can treat noise-induced hearing loss (Gedik et al., 2020). Kesici et al. (2018) performed permanent threshold shift noise-induced hearing loss modeling in rats and found that administering metformin by gavage before noise exposure resulted in hearing recovery in the experimental rats. The cochlear morphology and staining intensity of apoptotic factors were generally consistent with those of normal rats (blank control group) without noise exposure (Gedik et al., 2020). In 2022, *Nature* reported that the target of action of metformin is the activated AMPK (Ma et al., 2022). Activated AMPK can phosphorylate the key enzyme HMGR to inhibit cholesterol synthesis (Wang et al., 2018). Based on the results presented above, we speculated that metformin may prevent noise-induced hearing loss by inhibiting cholesterol synthesis through the activation of the AMPK pathway in auditory cells.

### 2.4 Hearing loss caused by ototoxic drugs may be related to disorders of intracellular cholesterol homeostasis in the cochlea

Hydroxypropyl-β-cyclodextrin (HPβCD) is a cholesterol chelator that has been shown to be effective in treating lipid metabolism disorders, such as Niemann-Pick disease (Yamada et al., 2021, 2022, 2023). However, it has been found to have a severe damaging effect on patients’ hearing. A phase I–II clinical trial of HPβCD in NPC1 patients was published in *The Lancet* in 2017, in which all participants (*n* = 14) experienced irreversible mid- to high-frequency hearing loss (Ory et al., 2017). Regardless of whether HPβCD was administered into the central or peripheral nervous system, experimental animals showed preferential loss of outer hair cells in the cochlea within hours of administration. Therefore, HPβCD is considered an ototoxic drug that targets the outer hair cells. Studies have found that HPβCD administration to mice with the outer hair cell electromotility protein prestin knocked out can alleviate HPβCD-induced outer hair cell death. Therefore, it is speculated that HPβCD may disrupt the distribution of cholesterol on the outer hair cell membrane through the prestin protein, which leads to damage to the outer hair cells, rather than by affecting the lateral membrane motor function of outer hair cells (Zhou et al., 2018). 3β-hydroxysterol Δ (24)-reductase (DHCR24) is a critical enzyme that catalyzes cholesterol biosynthesis in cells and can convert desmosterol to cholesterol (Zerenturk et al., 2013). Studies have demonstrated that, from the birth to the development of normal hearing in rats, DHCR24 is expressed in the cochlear hair cells and spiral ganglion neurons of newborn rats. Inhibiting the expression of DHCR24 increases reactive oxygen species and cleaved-caspase-3 expression and aggravates hair cell loss caused by *in vitro* cisplatin damage (Tian et al., 2019). This study suggested that normal cholesterol synthesis plays a protective role in cisplatin-induced ototoxicity (Tian et al., 2019). However, some studies have shown that inhibiting cholesterol synthesis has a protective effect against cisplatin ototoxicity (Min and Kim, 2007; Yu et al., 2010). Puerarin is an isoflavone compound with antioxidant and cholesterol-lowering effects; it is often used to treat neurodegenerative and cardiovascular diseases. A Korean study reported that Pueraria thunbergiana can reduce oxidative stress induced by cisplatin and protect auditory cells (Yu et al., 2010). The active ingredients extracted from Pueraria thunbergiana, irisolidone and Kakkalide, have cholesterol-lowering effects, with HMG-CoA as their target (Min and Kim, 2007). Therefore, we speculated that the protective effect of Pueraria thunbergiana against cisplatin-induced ototoxicity may be related to the inhibition of cholesterol synthesis.

### 2.5 Disorders of intracellular cholesterol homeostasis in cochlea may aggravate age-related hearing loss

Hypercholesterolemia has been reported by many researches as a risk factor for age-related hearing loss. However, excessively low cholesterol level also has harmful effect on auditory function. Malgrange et al. (2015) found that a decrease in blood cholesterol level is associated with the occurrence and development of age-related hearing loss. Recent studies have further indicated that the imbalance of cholesterol homeostasis within auditory cells may be a significant risk factor for age-related hearing loss. Sodero et al. (2023) reported a decreased level of cholesterol within cochlear hair cells during the aging process by comparing 2-month-old and 24-month-old C57BL/6J mice. Cholesterol 24-hydroxylase is an important enzyme regulating cholesterol efflux in the central nervous system. Immunofluorescence was utilized for the staining of CYP46A1 (cytochrome P450 family 46 subfamily A member 1) in cochlear hair cells and significant increased expression was found in 24-month-old mice, indicating that decreased cholesterol level in cochlear hair cells might be associated with increased cholesterol 24-hydroxylase expression. Significant decreases of cholesterol level, prestin expression and an increase in the DPOAE thresholds were found in cochlear outer hair cells of 2-month-old mice after four-week feeding with a 24-hydroxylase agonist. More importantly, the damage in outer hair cell and hearing loss caused by the 24-hydroxylase agonist could be alleviated after the co-administration of cholesterol analog—phytosterols. These results suggest that cholesterol homeostasis imbalance is a crucial cellular pathological feature during the aging process of cochlear hair cells in C57BL/6J mice. Rectifying the imbalance of cholesterol homeostasis within hair cells may delay the aging of auditory cells and alleviate hearing loss (Sodero et al., 2023).

Studies on elderly individuals with age-related hearing loss found that many food extracts can delay age-related hearing loss by affecting cellular cholesterol homeostasis, such as omega-3 (Tang et al., 2019; Kim et al., 2020; Rodrigo et al., 2021). Omega-3 is a type of polyunsaturated fatty acid that is primarily obtained through food absorption and is also known as an essential fatty acid. It has anti-inflammatory and antioxidant effects. Omega-3 maintains cellular homeostasis by inhibiting HMG-CoA reductase and reducing endogenous cholesterol synthesis (Honkura et al., 2019). Mammals lack omega-3 desaturase; so, omega-3 cannot be synthesized autonomously. The Fat-1 gene is derived from the nematode Caenorhabditis elegans and encodes a fatty acid desaturase that plays a key role in the synthesis of *n*-3 polyunsaturated fatty acids. Overexpression of this gene in mice increases the levels of 3-polyunsaturated fatty acids in tissues and organs. Using these transgenic mice (Fat-1 transgenic mice), Honkura et al. (2019) found that 13-month-old transgenic mice (C57BL/6N strain) fed with a normal diet showed a significant decrease in the threshold at 12 kHz by auditory brainstem response testing, indicating that increasing endogenous omega-3 can delay age-related hearing loss.

## 3 Outlook and speculation

**FIGURE 1:**
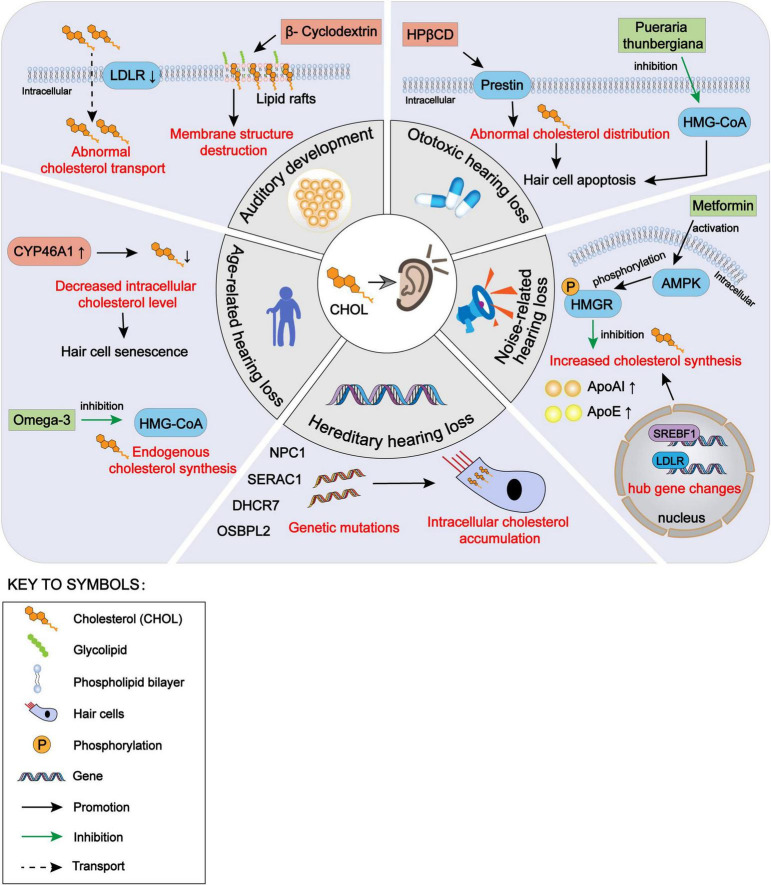
The impact of intracellular cholesterol homeostasis disorder within auditory cells on hearing, encompassing auditory development, hereditary hearing loss, noise-induced hearing loss, ototoxic hearing loss and age-related hearing loss. CHOL, cholesterol; LDLR, low-density lipoprotein receptors; NPC1, Niemann-Pick disease type C1; SERAC1, serine active site-containing protein 1; DHCR7, 7-dehydrocholesterol reductase; OSBPL2, oxysterol binding protein-like 2; CYP46A1, cytochrome P450 family 46 subfamily A member 1; HMG-CoA, 3-hydroxy-3-methyl glutaryl coenzyme A; HPβCD, hydroxypropyl-β-cyclodextrin; AMPK, adenosine 5′-monophosphate-activated protein kinase; HMGR, HMG-CoA reductase; ApoAI, apolipoprotein AI; ApoE, apolipoprotein E; SREBF1, sterol regulatory element-binding protein 1.

In this paper, we have reviewed the effects of cholesterol homeostasis disorders within auditory cells on the hearing development and various types of hearing loss, as well as the protective effect on the auditory function by restoring the balance of cholesterol homeostasis (Figure 1). Cholesterol is a natural agonist of the NLRP3 inflammasome (Sun et al., 2020). As intracellular cholesterol homeostasis is abnormal, it often triggers cell damage responses, such as inflammatory stimulation and oxidative stress. Therefore, it is essential to explore the regulation of the cholesterol homeostasis within cochlear hair cells. Some previous studies have evaluated the use of cholesterol metabolism-related therapeutic targets, such as HMG-CoA inhibitors and LXR agonists, in neurological diseases, especially optic nerve diseases. These reports are valuable to the exploration of the effects of cholesterol homeostasis disorder within the cochlear hair cells on the hearing loss. Many issues need to be explored in the future. First, due to the fine structure of the cochlea and technical limitations, few studies have been conducted on the normal level of cholesterol in the internal and external lymph fluids and on the change in cholesterol level in pathological conditions. Second, the transport methods of cholesterol in the cochlea from the peripheral blood and between hair cells and supporting cells need further exploration. Third, the mechanism underlying the change in cholesterol homeostasis within auditory cells in some types of hearing loss needs to be explored further. We suggest that future studies should explore the role of intracellular cholesterol homeostasis balance in hearing maintenance, as well as the role of intracellular cholesterol homeostasis imbalance in hearing loss for the prevention and treatment of hearing loss. In addition, further studies should be conducted on cholesterol synthesis, transport, absorption, and efflux within inner ear cells.

## Author contributions

JW: Conceptualization, Writing – original draft, Writing – review & editing. PJ: Writing – review & editing, Writing – original draft, Investigation. AZ: Writing – review & editing, Writing – original draft, Data curation. HH: Writing – review & editing, Funding acquisition. YS: Writing – review & editing, Funding acquisition. QW: Funding acquisition, Writing – review & editing. CF: Writing – review & editing. KC: Writing – review & editing. RD: Writing – review & editing. WH: Writing – review & editing. MX: Writing – review & editing, Conceptualization, Validation, Writing – original draft. BY: Writing – review & editing, Funding acquisition, Validation, Writing – original draft.
